# MicroRNAs as promising biomarkers in cancer diagnostics

**DOI:** 10.1186/2050-7771-2-19

**Published:** 2014-10-22

**Authors:** Prasun J Mishra

**Affiliations:** 1 US Department of Health and Human Services, National Cancer Institute, National Institutes of Health, Laboratory of Cancer Biology & Genetics, Bethesda, MD 20892, USA

**Keywords:** microRNA, Biomarker, Diagnostics, SNPs, miRSNP, Polymorphism, Circulating microRNAs, Exosomic, Tumor microenvironment, Colorectal cancer, Osteosarcoma, Clear cell renal cell carcinoma, miRNA variant, Metastasis

## Abstract

Cumulating data suggest that small noncoding-RNAs such as microRNAs (miRNAs) can be utilized as potential biomarkers for the diagnosis and prognosis of a variety of diseases such as cancer, neurological disorders, cardiovascular disease and Type-II diabetes, etc. MiRNAs can be utilized not only for monitoring of treatments but also for patient stratifications. The Tenth Annual miRNA as Biomarkers and Diagnostics conference, 2014, organized in Boston, MA, was primarily focused on recent advancements in the field of miRNA in the early detection of disease, monitoring tumor growth/progression and its potential for precision medicine. This article summarizes findings presented in the miRNA biomarker as cancer diagnostics session. The overarching projections from this and other sessions were that miRNAs are now well established as regulators of tumorigenesis and can be utilized not only as potential biomarkers for the diagnosis and prognosis of a disease but also are useful in patient stratifications and treatment response.

## Findings

The data presented at the microRNA (miRNA) biomarker as cancer diagnostics session clearly suggested that miRNA profiling has been useful in identifying predictive miRNA signatures associated with tumor growth/progression of various cancer types such as pancreatic cancer, colorectal cancer, osteosarcoma as well as clear cell renal cell carcinoma. Changes of specific miRNAs can be detected in tissues utilizing a slide-based staining assay and from circulating tumor cells from the blood sample as well as in the urine, presenting a noninvasive way to detect miRNAs. Furthermore, detection of exosomic miRNAs in the tumor microenvironment may provide better tools for development of new personalized treatments for cancer patients. Moreover, detection of miRNA‒polymorphisms and miRNA variants may help further improve diagnosis, treatment and prognosis in patients and has profound implications in the fields of pharmacogenomics and precision medicine.

Small noncoding-RNAs such as microRNAs (miRNAs) are emerging as prominent disease associated biomarkers and can be utilized not only for patient stratifications but also for monitoring of treatments (i.e. for diagnosis and prognosis). To discuss this, The Tenth Annual microRNA as Biomarkers and Diagnostics conference (March 17–18, 2014), organized in Boston, MA, USA brought over 100 representatives from academia, government, clinical laboratories and industry and comprised of 20 presentations (101). The conference broadly covered topics in the area of 1) miRNA biomarkers in drug development; 2) miRNA biomarkers as cancer diagnostics; and 3) miRNA as disease biomarkers (101). This article summarizes the session regarding *miRNA biomarker as cancer diagnostics*.

## Chairperson’s opening remarks

Prasun J. Mishra, Ph.D., (US Department of Health and Human Services, National Cancer Institute, National Institutes of Health) opened the session by describing the need for predictive biomarkers in cancer progression/prognosis and why miRNAs are promising candidates in predicting drug response. MiRNAs are small non-coding RNAs, about 21–25 nucleotides in length, that are emerging as a new class of biomarkers. The cancer research field has been at the forefront of advancing miRNA biomarker field for over a decade now. This has been made possible, not only due to the availability of the tumor tissue samples in the clinic, but also recent advancements in the field of miRNA detection and sequencing. MiRNAs are now well established as regulators of tumorigenesis. In cancer, miRNA expression variations are evident across different stages of cancer progression [1]. In malignancy, miRNA expression is altered (overexpressed/underrepresented) [2]. Overexpressed miRNAs in cancer may act like oncogenes by down-regulating tumor suppressor genes. Vice versa is also true, down modulated tumorsuppressor-like miRNAs result in upregulation of oncogenes there by functioning as tumor suppressors. Hence, in a malignant tumor, the oncogenic miRNAs are upregulated and tumor suppressor miRNAs are downregulated. These miRNAs can be exploited as potential biomarkers. miRNAs are also tissue specific and may be unique identifiers of tumor type and its origin [3]. An increasing number of miRNAs are now identified and utilized as prognostic miRNAs to predict drug response.

The importance and the uniqueness of this session were also emphasized. This particular session was designed to cover recent advancements in the miRNA as biomarker and cancer diagnostics field. The speakers discussed utility of non-coding RNA variants to precision medicine (Prasun Mishra) as well as role of miRNA in tumor microenvironment (Muller Fabbri). The session also highlighted utility of miRNAs profiling in monitoring tumor growth/progression of pancreatic cancer (Lorenzo Sempere), colorectal cancer (Jingfang Ju), circulating osteosarcoma cells (Benjamin Ory) and clear cell renal cell carcinoma (Huiqing Wu) and its utility as a biomarker for precision medicine.

## MicroRNA variants as molecular diagnostics and prognostic tools

Prasun J. Mishra (US Department of Health and Human Services, National Cancer Institute, National Institutes of Health) described recent advances in the field of miRNA polymorphisms and their implications to precision medicine. Recent advancements in microRNA field suggest that genetic variation or polymorphisms present in the miRNA pathway are associated with the prognosis/progression of diseases and drug responses and are emerging as powerful tools to study biology of diseases [4-6]. MicroRNA polymorphisms (miR‒polymorphisms) can be defined as polymorphisms (SNPs, chromosomal changes, epigenetic defects, mutations, alterations and variations) that may potentially interfere with miRNA‒mediated regulation of cellular functions and can be present not only in the miRNA target gene but also in pri‒, pre‒, mature‒miRNA sequences, in the genes involved in miRNA biogenesis and in miRNA cis‒regulatory elements (e.g. promoter) [7]. A polymorphism in mature miRNAs may affect expression of several genes and have serious consequences, whereas a polymorphism in miRNA target site may be more target and/or pathway specific [7]. For example, mir-24 regulates expression of anti-cancer drug methotrexate (MTX) target dihydrofolate reductase (DHFR) by binding to its 3′UTR; a SNP in DHFR 3′ UTR results in loss of the miR-24 function (miRSNP), an increase in DHFR expression and MTX-chemoresistance [4]. Thus, in addition to gene amplification causing DHFR overexpression [8], loss of miRNA function due to a miRSNP represents a new mechanism of target overexpression and drug resistance [8] and establishes a miRNA-drug resistance link [8]. In a follow up report, miR-24 was demonstrated to act as a p53-independent tumor suppressor miRNA, and loss of mir-24 function due to the miRSNPs not only confers drug resistance but also imparts a growth advantage to immortalized cells and induces neoplastic transformation [6]. Thus, recent advances in the miRNA field have led to the understanding of entirely new mechanisms of cellular transformation and drug-resistance caused by loss of miRNA function by an miRNA variant (miRSNP/miR-polymorphisms) [4-6]. Moreover, the discovery of the role of miRNA in drug resistance and miR‒polymorphisms to predict drug response has led to the development of a new field in biomedical science called miRNA pharmacogenomics, a study of the miRNAs and miR‒polymorphisms affecting expressions of drug target genes, to predict drug behavior and to improve drug efficacy. Detection of miRNA‒polymorphisms can potentially improve diagnosis, treatment and prognosis in patients and has profound implications in the fields of pharmacogenomics and precision medicine [9].

## Role of exosomic miRNAs in the tumor microenvironment

Muller Fabbri (Pediatrics and Molecular Microbiology & Immunology, University of Southern California Keck School of Medicine, Children’s Hospital Los Angeles) described role of exosomic miRNA in tumor microenvironment and it’s implication to precision medicine. Cancer is a genetic disease and an increasing evidence indicates that not only cancer cells but also surrounding cells, forming the so-called “tumor microenvironment”, play a key role in cancer biology [10]. MiRNAs are also secreted by cancer cells in the surrounding tumor microenvironment, within microvesicles (called exosomes). Exosome-contained miRNAs (namely, miR-21 and miR-29a) can be engulfed by the immune cells surrounding cancer cells and they can bind to Toll-like Receptor 8 (TLR8) present in the immune cells [10]. As a consequence, TLR8 is activated and the immune cells release interleukin-6 (IL-6) and tumor necrosis factor alpha (TNF-alpha), which increase tumor growth and metastatic potential [11]. A better understanding of this novel mechanism of miRNA action will contribute to the development of new personalized treatments for cancer patients [10].

## Tissue slide-based microRNA diagnostics

Lorenzo F. Sempere (Center for Personalized Medicine, Van Andel Research Institute) described a slide based miRNA diagnostics tool for pancreatic cancer. MiRNA diagnostics is an emerging area of prognostic and predictive indicators in cancer medicine. Dr. Sempere presented his study focused on the role of miRNAs in initiation and progression of pancreatic cancer [12]. Recent studies in the pancreatic cancer field have shown that there are changes in the levels of miRNAs between normal and tumor tissues [13]. Tissue slide-based assays are routine diagnostic procedures in clinical laboratories to guide treatment options. This study implemented a fully automated tissue slide-based staining assay in a CLIA-certified environment utilizing in situ hybridization to identify specific miRNAs alterations in the pancreas of mouse models that develop pancreatic cancer. Since pancreatic tissue is composed of different cell types and only some of these cells are susceptible to develop cancer, it is important to know if these miRNA changes occur within the cancer-prone cells. Hence this study aimed to determine whether neutralizing these miRNA changes affect the growth and survival properties of the cancer cells. Potential utility of the assay were described in measuring levels and cellular compartment of miRNA expression in solid tumors and potential diagnostic applications of salient miRNA examples (including miR-21 and miR-34a) were discussed [12].

## MicroRNAs as biomarkers in colorectal cancer

Jingfang Ju (Translational Research, Pathology, Stony Brook University) described recent developments in the area of miRNAs as biomarkers in colorectal cancer. 5-Fluorouracil based chemotherapy (e.g. FOLFOX) is the standard chemotherapeutic regimen for treating advanced stage colorectal cancer. Chemoresistance is the major reason for the failure of treating advanced colorectal cancer [14]. MiRNA based therapeutics, diagnosis and prognosis may emerge in the near future to benefit cancer patients. Several miRNAs were found to be associated (miR-192, miR-215, miR-140, miR-129, let-7, miR-181b, miR-200 s) with chemoresistance by regulating key cell death pathways such as apoptosis and autophagy. Several important miRNA were described that regulate targets such as Bcl2, thymidylate synthase, dihydrofolate reductase, histone deacetylase, and E2F. For example, miR-215 was identified to suppress the expression of both thymidylate synthase and dihydrofolate reductase [15]. In addition, the expression of miR-215 was directly regulated by p53. The expression of miR-215 was significantly associated with colorectal cancer patient survival. Another miRNA, miR-140, was found to modulate chemosensitivity by suppressing HDAC4 expression, and the levels of miR-140 and miR-215 were elevated in colon cancer stem cells [16]. Furthermore, miR-194 was identified to regulate BMI-1 protein expression (BMI-1 is involved in epithelial to- mesenchymal transition) [17]. Moreover, miR-502 regulates autophagy in colon cancer by targeting Rab1B [18]. Taken together, these miRNAs can be utilized in predicting patient’s prognosis and survival.

## MicroRNA profiling of circulating tumor cells from osteosarcoma

Benjamin Ory (Therapy Primary Bone Tumor, Nantes Medical School, INSERM) described a miRNA profiling method [19] of circulating tumor cells. Through the isolation of the rare circulating tumor cells (CTCs) in the blood stream, Dr. Ory and his group uncovered some basic functional roles of the miRNAs in the regulation of pathways involved in the cellular migration and invasion, responsible for the metastasis dissemination of osteosarcoma (unpublished). This study identified a panel of new candidate miRNAs as predictive biomarkers for osteosarcoma metastasis from the analysis of blood samples.

## A miRNA signature to predict metastatic clear cell renal cell carcinoma

Huiqing Wu (Pathology, City of Hope National Medical Center, Beckman Research Institute) described a miRNA signature to predict clear cell renal carcinoma. Using frozen tissue cohorts, microarray technology and risk score method, Dr. Wu’s group identified a 4-miRNA signature associated with clear cell renal cell carcinoma (ccRCC) metastasis and prognosis [20,21]. The signature can be validated on a formalin-fixed paraffin-embedded (FFPE) tissue-specific and RT-PCR based assay. The gene signature was further validated in an FFPE tissue cohort of 222 cases of primary ccRCC, with an overall sensitivity and specificity of 70% and 76%, respectively. The sensitivity and specificity for predicting metastasis from stage I and II patients were 59% and 74%, respectively. The values for stage III patients were 80% and 83%. The signature was associated with the patient’s cancer-specific survival and can be utilized as a predictive biomarker.

## Conclusion

The overarching projections from this and other sessions were that miRNAs are now well established as regulators of tumorigenesis and can be utilized not only as potential biomarkers for the diagnosis and prognosis of a disease but also are useful in patient stratifications and treatment response [22]. The data presented at this session clearly suggested that miRNAs profiling has been useful in identifying predictive miRNA signatures associated with tumor growth/progression of various cancer types such as pancreatic cancer, colorectal cancer, osteosarcoma as well as clear cell renal cell carcinoma. Changes of specific miRNAs can be detected in tissues utilizing a slide-based staining assay and from circulating tumor cells from the blood sample as well as in the urine, presenting a noninvasive way to detect miRNAs. Furthermore, detection of exosomic miRNAs in the tumor microenvironment may provide better tools for development of new personalized treatments for cancer patients. Moreover, detection of miRNA‒polymorphisms and miRNA variants may help further improve diagnosis, treatment and prognosis in patients and has profound implications in the fields of pharmacogenomics and precision medicine.

## Abbreviations

miRNA: microRNA; UTR: Untranslated region; miRSNP/mir-polymorphism: A SNP or polymorphism that results in loss of a miRNA function; ccRCC: Clear cell renal cell carcinoma; FFPE: Formalin-fixed paraffin-embedded; RT-PCR: Real time PCR; CTCs: Circulating tumor cells; FOLFOX: A chemotherapy regimen to treat colorectal cancer, it includes FOL– Folinic acid (leucovorin), F – Fluorouracil (5-FU) and OX – Oxaliplatin (Eloxatin); TLR8: Toll-like receptor 8; IL-6: Interleukin-6; TNF-alpha: Tumor necrosis factor alpha; DHFR: Dihydrofolate reductase; MTX: Methotrexate.

## Competing interests

The authors declare that they have no competing interests.

## Authors’ information

Dr. Mishra is a scientist at National Cancer Institute (NCI), National Institutes of Health (NIH). Prior to NCI, he worked at Rutgers Cancer Institute of New Jersey, Robert Wood Johnson Medical School, Rutgers University, for seven years, where his research focus was to identify novel mechanisms of drug resistance, identify mechanism of actions of new drugs, and design novel drugs/combinations to facilitate anticancer drug development. He received his Ph.D. from Rutgers University in Cellular and Molecular Pharmacology with university’s highest honors. In 2008 Dr. Mishra joined the US Department of Health and Human Services, NCI, NIH, where his research focus has primarily been to identify and exploit “druggable” early genetic events, driving cancer progression, using integrated genomics and high-throughput screening, to design a more effective anticancer therapy. His discoveries have led to the development of new and viable strategies of prevention, diagnosis and treatment of cancer patients in the clinic.
